# 高效液相色谱-串联质谱法测定大气细颗粒物中7种轮胎抗氧化剂及其醌类氧化物

**DOI:** 10.3724/SP.J.1123.2025.09023

**Published:** 2026-06-08

**Authors:** Feng LIU, Binbin TANG, Liyuan WU, Lixin SONG, Min ZHANG, Min HE

**Affiliations:** 包头市疾病预防控制中心（市卫生监督所），内蒙古 包头 014030; Baotou Center for Disease Control and Prevention （Municipal Health Supervision Institute），Baotou 014030，China

**Keywords:** 高效液相色谱-串联质谱法, 大气细颗粒物, 对苯二胺类化合物, 对苯二胺醌类氧化物, high performance liquid chromatography-tandem mass spectrometry （HPLC-MS/MS）, fine particulate matter （PM_2.5_）, *p*-phenylenediamine compounds （PPDs）, *p*-phenylenediamine quinones （PPD-Qs）

## Abstract

建立了一种测定大气细颗粒物（PM_2.5_）中7种对苯二胺类化合物（*p*-phenylenediamines， PPDs）及其醌类氧化物（PPD-Qs）的高效液相色谱-串联质谱方法。样品通过90 mm石英纤维滤膜采集后，采用含1%（体积分数）氨水和20 μmol/L谷胱甘肽的乙腈提取，QuEChERS方法净化及氮吹浓缩后，以5 mmol/L甲酸铵（含0.1%甲酸）和甲醇为流动相，通过Waters ACQUITY UPLC HSS T_3_（100 mm×2.1 mm，1.8 μm）色谱柱分离，在电喷雾正离子源多反应监测模式下检测，内标法定量。结果表明，各目标物在0.05~20.0 ng/mL范围内线性关系良好（相关系数*r*均大于0.999），方法检出限和方法定量限分别为0.003~0.07 pg/m^3^和0.01~0.2 pg/m^3^。在低、中、高3个加标水平下，各目标物的回收率为66.3%~119.0%，相对标准偏差为1.5%~13.2%（*n=*6）。应用该方法检测包头市2025年1、2月的PM_2.5_样品，7种PPDs和5种PPD-Qs被检出，质量浓度范围分别为2.26~251.2 pg/m^3^和2.36~105.4 pg/m^3^。该方法简便、环保、准确，适用于PM_2.5_中PPDs和PPD-Qs的快速定量分析。

## Abstract (en)

*p*-Phenylenediamine compounds （PPDs） are widely used as anti-aging agents and antioxidants in rubber industry. However， environmental concerns have arisen regarding the toxicity of their quinone derivatives （PPD-Qs）. Fine particulate matter （PM_2.5_） serves as a crucial vector enabling PPDs and PPD-Qs to enter the human body via respiratory exposure pathway. Therefore， accurate monitoring of PPDs and PPD-Qs in PM_2.5_ is essential. In this research， a high performance liquid chromatography-tandem mass spectrometry （HPLC-MS/MS） approach was established to quantify seven PPDs and seven PPD-Qs in PM_2.5_. Samples were collected on quartz fiber filters with a diameter of 90 mm. The filter was cut into strips and subsequently spiked with 2 ng of 6PPD-d_5_ and 6PPD-Q-d_5_ internal standards. The target compounds were extracted with 10 mL of acetonitrile containing 1% （volume fraction） ammonia solution and 20 μmol/L glutathione （GSH）， and purified via a modified QuEChERS pretreatment method. Specifically， after salting out with 0.5 g of NaCl and 1.0 g of MgSO_4_， the supernatant was subjected to dispersive solid-phase extraction for cleanup. In this process， 0.5 g of MgSO_4_， 100 mg of octadecylsilane （C_18_）， and 100 mg of *N*-propylethylenediamine （PSA） were employed as sorbents. Subsequently， 5 mL of the cleaned-up supernatant was evaporated to near dryness and then redissolved in 0.2 mL of a methanol-water mixture （1∶1， volume ratio） for instrumental determination. The compounds were separated on a Waters ACQUITY UPLC HSS T_3_ column（100 mm×2.1 mm，1.8 μm） with mobile phases consisting of a 5 mmol/L ammonium formate aqueous solution containing 0.1% formic acid and methanol. Identification and quantification of the compounds were carried out under positive electrospray ionization in multiple reaction monitoring mode. The results indicated that all analytes exhibited good linear relationships within the range of 0.05-20.0 ng/mL， with correlation coefficients （*r*） exceeding 0.999. The method detection limits （MDLs） and method quantification limits （MQLs） of the compounds were within the ranges of 0.003-0.07 pg/m^3 ^and 0.01-0.2 pg/m^3^， respectively. At three spiked levels of low， medium and high， the recoveries of the compounds were in the range of 66.3% to 119.0% ， and the relative standard deviations （RSDs） ranged from 1.5% to 13.2% （*n*=6）. Ultimately， the developed method was employed for the analysis of PPDs and PPD-Qs in PM_2.5_ samples collected from Baotou during January and February 2025. The results indicated that seven PPDs and five PPD-Qs were detected， with mass concentrations ranging from 2.26 to 251.2 pg/m^3^ and 2.36 to 105.4 pg/m^3^， respectively. The proposed method is characterized by its simplicity， environmental friendliness and accuracy， making it suitable for the rapid quantitative analysis of PPDs and PPD-Qs in PM_2.5_.

对苯二胺类化合物（*p*-phenylenediamines，PPDs）是橡胶工业中常见的防老剂和抗氧化剂，被广泛用于汽车轮胎等橡胶制品中，主要包括*N*-（1，3-二甲基丁基）-*N′*-苯基对苯二胺（6PPD）、*N*-环己基-*N′*-苯基对苯二胺（CPPD）及*N*-异丙基-*N′*-苯基-1，4-苯二胺（IPPD）等。这些化合物通过轮胎磨损颗粒的扩散、迁移和地表径流的冲刷等，广泛分布在大气^［1，2］^、水体^［3，4］^和土壤^［5］^等各种环境介质中。在臭氧等条件下，释放至环境中的PPDs还会转化为其醌类氧化物PPD-Qs^［6］^。2021年，Tian等^［7］^发现6PPD-Q能够导致太平洋银鲑的急性死亡。后续实验证明，PPD-Qs还会引起哺乳动物肝肾^［8］^、呼吸系统^［9，10］^及神经系统^［11，12］^等损害。目前，已在人体血液、尿液、母乳、脑脊液等生物样本中检测出该类物质^［13-15］^。PPDs及PPD-Qs的环境行为和潜在毒性已成为社会关注的热点问题。

大气细颗粒物（fine particulate matter，PM_2.5_）可吸附和载带多种有机化合物^［16］^，是PPDs和PPD-Qs经呼吸暴露途径进入人体的重要载体。因此，准确监测PM_2.5_中PPDs和PPD-Qs的污染状况具有重要意义。目前，PM_2.5_中该类化合物的检测技术主要包括超高效液相色谱-质谱法（UHPLC-MS）^［17-20］^、超高效液相色谱-四极杆-静电场轨道阱-质谱法（UPLC-Q Orbitrap MS）^［21］^和高效液相色谱-四极杆-飞行时间质谱联用法（HPLC-Q-TOF-MS）^［22］^等。样品的前处理技术主要包括超声波辅助萃取和氮吹浓缩法^［17-22］^。Zhang等^［17］^建立的前处理方法中分别使用4 mL正己烷、4 mL丙酮和3 mL正己烷-丙酮（1∶1，体积比）提取目标物。Wang等^［21］^使用5 mL二氯甲烷提取2次后，再用5 mL乙腈提取1次，提取总时长45 min。Abudumutailifu等^［22］^使用10 mL甲醇提取PM_2.5_中的PPDs和PPD-Qs，提取时长30 min，该提取过程重复3次。Wang等^［23］^先后以5 mL二氯甲烷、5 mL乙腈和5 mL去离子水作为提取溶剂，分3次提取目标物，提取时长90 min。综上，目前大多数PM_2.5_中PPDs和PPD-Qs的提取采用多溶剂提取法，并存在提取溶剂用量多（≥11 mL）、耗时长（≥45 min）和步骤繁琐（提取次数≥3）等不足。

基于此，本研究尝试采用单一溶剂萃取法，并建立了一种一步式QuEChERS-高效液相色谱-串联质谱方法，用于PM_2.5_中7种PPDs及其醌类氧化物的检测。

## 1 实验部分

### 1.1 仪器和试剂

TH-150C智能中流量PM_2.5_采样器（武汉市天虹仪表有限责任公司）；LCMS-8030高效液相色谱-串联四极杆质谱仪（日本岛津公司）；MULTIFUGE XIR高速冷冻离心机（美国Thermo Fisher公司）；Scout百分之一电子天平（美国奥豪斯仪器有限公司）；Multi Reax涡旋混匀器（德国Heidolph公司）；EQ 7000超纯水机（德国默克公司）；MFV-24氮吹仪（广州得泰仪器科技有限公司）；KH-500E超声波清洗器（昆山禾创超声仪器有限公司）。

7种PPDs和7种PPD-Qs标准溶液及标准品：CPPD、*N*-（1，4-二甲基戊基）-*N*′-苯基对苯二胺（7PPD）、*N，N′*-双（1，4-二甲基戊基）-对苯二胺（77PD）、*N，N′*-二（邻甲苯基）对苯二胺（DTPD）以乙腈为溶剂；6PPD、IPPD、*N，N′*-二苯基-对苯二胺（DPPD）以甲苯为溶剂；6PPD-Q以甲醇为溶剂；*N*-（1，4-二甲基戊基）-*N′*-苯基对苯二胺醌（7PPD-Q）（纯度98%）；*N，N′*-双（1，4-二甲基戊基）-对苯二胺醌（77PD-Q）、*N*-环己基-*N′*-苯基对苯二胺醌（CPPD-Q）、*N，N′*-二（邻甲苯基）对苯二胺醌（DTPD-Q）、*N，N′*-二苯基-对苯二胺醌（DPPD-Q）以二甲基亚砜为溶剂；*N*-异丙基-*N′*-苯基-对苯二胺醌（IPPD-Q）以丙酮为溶剂。考虑到PPDs和PPD-Qs各自具有相类似的分子结构，为节约实验成本，使用了2种同位素内标，分别为6PPD-d_5_（以丙酮为溶剂）和6PPD-Q-d_5_（以乙腈为溶剂）。除DPPD-Q标准溶液的质量浓度为50 μg/mL外，其他标准溶液及同位素内标的质量浓度均为100 μg/mL。以上标准溶液及标准品均购于天津阿尔塔科技有限公司。甲醇和乙腈（色谱纯，美国Fisher公司）；氨水、无水硫酸镁和氯化钠（分析纯，国药集团化学试剂有限公司）；十八烷基硅烷键合硅胶（C_18_）（6 nm，天津博纳艾杰尔公司）；*N*-丙基乙二胺（PSA，上海迪马科技有限公司）；谷胱甘肽（还原型）（≥98%，上海阿拉丁生化科技股份有限公司）；石英纤维滤膜（90 mm，英国Cytiva Whatman）；实验用水为超纯水。

### 1.2 标准溶液及系列基质标准工作液的配制

首先，准确称取7PPD-Q标准品，用甲醇溶解并定容，配制成质量浓度为100 μg/mL的标准储备溶液。然后，分别移取DPPD-Q标准溶液200 μL和其余13种单一标准溶液各100 μL于10 mL容量瓶中，用甲醇定容，配制成1.0 μg/mL的混合标准溶液。分别移取2种同位素内标标准溶液各100 μL于10 mL容量瓶中，用甲醇定容，配制成1.0 μg/mL的混合内标溶液。配制好的PPDs和PPD-Qs混合标准溶液及混合内标溶液于-20 ℃避光储存。

系列基质标准工作液的制备：取整张空白采样滤膜，剪碎后放入50 mL离心管。分别加入20、40、80 μL质量浓度为1.0 ng/mL的混合标准溶液，20、40、80 μL质量浓度为10 ng/mL的混合标准溶液，20、40、80 μL质量浓度为100 ng/mL的混合标准溶液，并分别加入20 μL质量浓度为100 ng/mL的混合内标标准溶液。然后完全按照样品前处理方法进行处理，最终获得质量浓度为0.05、0.1、0.2、0.5、1.0、2.0、5.0、10.0和20.0 ng/mL的系列标准工作液，内标质量浓度为5.0 ng/mL。

### 1.3 样品采集及前处理

使用中流量空气采样器采集PM_2.5_样品，采样地点为内蒙古包头市青山区包头医学院第二附属医院和东河区医院社区卫生服务中心楼顶，采样时间为2025年1、2月，每月连续采样7天（10~16日），每天采样时间不少于20 h，采样流量为100 L/min。共收集28份PM_2.5_样品，于-20 ℃冰箱中储存。采样前，将石英纤维滤膜用锡箔纸包好，置于400 ℃的马弗炉中烘烤4 h，去除有机物杂质。将恒量后的滤膜放入滤膜保存盒中备用。

取整张采样滤膜，剪碎后放入50 mL离心管，依次加入20 μL质量浓度为100 ng/mL的混合内标、10 mL 含1%（体积分数）氨水和20 μmol/L还原型谷胱甘肽的乙腈，冰浴超声提取30 min。然后在离心管中加入1.0 g无水硫酸镁和0.5 g氯化钠，涡旋1 min，4 ℃下9 000 r/min离心5 min。将上清液转移至含有0.5 g无水硫酸镁、100 mg C_18_和100 mg PSA的净化管中，涡旋1 min，4 ℃下9 000 r/min离心5 min。移取5.0 mL上清液至另一干净的15 mL离心管中。在40 ℃下氮气浓缩至近干，残渣用0.2 mL 50%甲醇水复溶，涡旋1 min，离心后供上机检测。需要说明的是，由于实验条件限制，本研究实际采用1/8样品滤膜进行检测。

### 1.4 仪器条件

色谱条件 色谱柱：Waters ACQUITY UPLC HSS T_3_（100 mm×2.1 mm，1.8 μm），柱温40 ℃，流动相A为5 mmol/L甲酸铵（含0.1%甲酸），流动相B为甲醇，流速为0.35 mL/min，进样量20 μL。梯度洗脱程序：0~1.0 min，90%A~5%A；1.0~8.0 min，5%A；8.0~8.1 min，5%A~90%A；8.1~11.0 min，90%A。

质谱参数 电喷雾离子源正离子模式（ESI^+^），多反应监测模式（MRM）扫描检测，脱溶剂温度250 ℃，接口电压4.5 kV，加热块温度400 ℃，雾化气流量3.0 L/min，干燥气流量15.0 L/min，其他质谱参数见表1。

**表1:** 7种PPDs、7种PPD-Qs和2种同位素内标的保留时间及质谱参数

Compound name	Abbr.	Retention time/min	Precursor ion （*m/z*）	Product ions （*m/z）*	Q1 Pre Bias/V	Collision energies/V	Q3 Pre Bias/V
*N*-Isopropyl-*N′*-phenyl-*p*-phenylenediamine （*N*-异丙基-*N′*-苯基-1，4-苯二胺）	IPPD	4.694	227.0	184.1^*^，107.0	-15，-12	-17，-43	-19，-11
*N*-cyclohexyl-*N′*-phenyl-1，4-benzenediamine （*N*-环己基-*N′*-苯基对苯二胺）	CPPD	5.092	267.1	184.0^*^，129.9	-14，-11	-22，-42	-20，-26
*N*-（1，3-Dimethylbutyl）-*N′*-phenyl-*p*-phenylenediamine （*N*-（1，3-二甲基丁基）-*N′*-苯基对苯二胺）	6PPD	5.283	269.3	184.1^*^，107.0	-15，-15	-20，-51	-13，-22
*N*-（1，4-Dimethylpentyl）-*N′*-phenylbenzene-1，4-diamine （*N*-（1，4-二甲基戊基）-*N′*-苯基对苯二胺）	7PPD	5.491	283.1	184.1^*^，93.0	-12，-12	-20，-36	-13，-20
*N*，*N*-Bis（1，4-dimethylpentyl）-*p*-phenylenediamine （*N，N′*-双（1，4-二甲基戊基）-对苯二胺）	77PD	5.653	305.2	135.0^*^，206.1	-16，-17	-34，-20	-29，-24
*N*，*N′*-Diphenyl-*p*-phenylenediamine （*N*，*N*-二苯基-对苯二胺）	DPPD	6.052	261.0	184.0^*^，107.0	-11，-11	-32，-47	-20，-21
*N*，*N′*-Bis（methylphenyl）-1，4- benzenediamine（*N，N′*-二（邻甲苯基）对苯二胺）	DTPD	6.723	289.1	198.0^*^，183.1	-15，-15	-26，-33	-20，-18
*N*-Isopropyl-*N′*-phenyl-*p*-phenylenediamine quinone（*N*-异丙基-*N′*-苯基-对苯二胺醌）	IPPD-Q	5.543	257.0	215.0^*^，187.0	-11，-14	-17，-28	-24，-20
*N*，*N′*-Diphenyl-*p*-phenylenediamine quinone（*N，N′*-二苯基-对苯二胺醌）	DPPD-Q	5.827	291.0	263.1^*^，235.0	-15，-12	-22，-30	-14，-24
*N*，*N′*-Bis（methylphenyl）-1，4-benzenediamine quinone（*N，N′*-二（邻甲苯基）对苯二胺醌）	DTPD-Q	6.058	318.9	212.0^*^，184.0	-18，-14	-19，-25	-14，-22
*N*-Cyclohexyl-*N′*-phenyl-1，4-benzenediamine quinone （*N*-环己基-*N′*-苯基对苯二胺醌）	CPPD-Q	6.162	297.1	187.0^*^，98.0	-16，-12	-32，-25	-19，-25
*N*-（1，3-Dimethylbutyl）-*N′*-phenyl-*p*-phenylenediamine quinone （*N*-（1，3-二甲基丁基）-*N′*-苯基-对苯二胺醌）	6PPD-Q	6.231	299.0	214.8^*^，241.0	-13，-16	-19，-33	-25，-17
*N*-（1，4-Dimethylpentyl）-*N′*-phenylbenzene-1，4-diamine quinone （*N*-（1，4-二甲基戊基）-*N′*-苯基对苯二胺醌）	7PPD-Q	6.514	313.1	215.0^*^，187.0	-16，-17	-19，-30	-24，-20
*N，N′*-Bis（1，4-dimethylpentyl）-*p*-phenylenediamine quinone （*N，N′*-双（1，4-二甲基戊基）-对苯二胺醌）	77PD-Q	7.335	335.2	237.1^*^，138.9	-18，-19	-21，-32	-17，-28
*N*-（1，3-Dimethylbutyl）-*N′*-phenyl-*p*-phenylenediamine-d_5_ （*N*-（1，3-二甲基丁基）-*N′*-苯基对苯二胺-氘5）	6PPD-d_5_	5.260	274.2	189.1^*^，107.0	-15，-15	-23，-50	-13，-23
*N*-（1，3-Dimethylbutyl）-*N′*-phenyl-*p*-phenylenediamineQuinone-d_5_（*N*-（1， 3-二甲基丁基）-*N′*-苯基-对苯二胺醌-氘5）	6PPD-Q-d_5_	6.208	304.1	220.0^*^，192.0	-16，-10	-20，-32	-24，-20

* Quantitative ion.

### 1.5 质量控制与保证

为确保目标分析物检测的准确性，样品采集过程中设置现场样品空白（全程序空白），检测分析中设置试剂空白，以监测采样和实验中是否引入外源性污染。参照中国疾病预防控制中心环境与健康相关产品安全所编制的《空气污染（雾霾）对人群健康影响监测与防护工作手册》（2022），试剂空白的检测结果低于方法检出限，现场样品空白的检测值低于方法定量限，并在计算实际样品检测结果时予以扣除。

### 1.6 数据统计与分析

采用Lab Solutions 5.91软件（日本岛津公司）对原始数据进行处理分析。采用SPSS 26进行数据统计分析，对未检出的数据按1/2检出限替代。

## 2 结果与讨论

### 2.1 仪器条件优化

首先，采用ESI^+^模式进行一级母离子扫描，得到各目标物的母离子［M+H］^+^，然后进行子离子扫描，扫描质量范围（*m/z*）为10~400，选择响应强度较高和稳定性较好的2个离子分别作为定量和定性离子。因PPDs的广泛适用性，需对溶剂空白进行严格考察^［24］^。经验证，试剂空白和样品空白均不存在明显的干扰。最终优化的质谱参数见表1。

其次，考察了有机相和水相对目标物色谱行为的影响。对于有机相，实验发现采用甲醇时，大部分PPD-Qs的响应强度均明显高于乙腈。对于水相，分别比较了0.1%甲酸水、2 mmol/L甲酸铵（含0.1%甲酸）、5 mmol/L甲酸铵（含0.1%甲酸）和10 mmol/L甲酸铵（含0.1%甲酸），发现水相中添加甲酸铵能够明显增加PPD-Qs的响应强度，5 mmol/L甲酸铵（含0.1%甲酸）明显优于0.1%甲酸水，进一步增加甲酸铵的浓度无显著效果。因此，选择5 mmol/L甲酸铵（含0.1%甲酸）-甲醇为流动相。此外，观察到CPPD在T_3_色谱柱上的响应强度明显高于C_18_色谱柱，故选用T_3_色谱柱。最终优化的色谱条件见1.4节，在该条件下各目标物响应强度、分离效果和色谱峰峰形均较好（图1）。

**图1:**
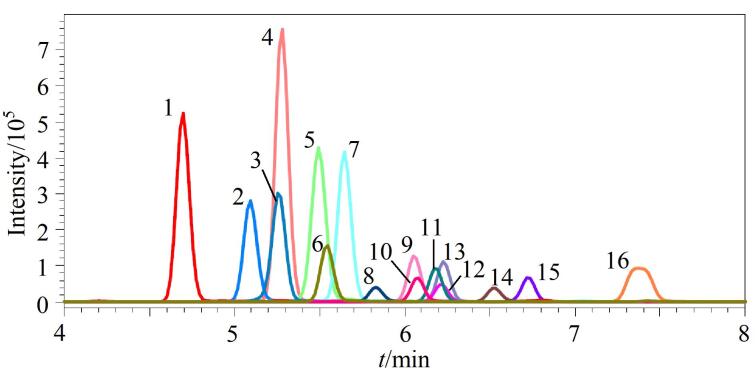
7种PPDs、7种PPD-Qs（10 ng/mL）和2种同位素内标（5 ng/mL）的MRM色谱图

### 2.2 样品前处理优化

#### 2.2.1 提取溶液

乙腈作为一种非质子中等极性溶剂，既能与亲水溶剂相溶，也能够与许多疏水溶剂相溶^［25］^，考虑使用乙腈作为提取试剂。提取溶液的pH影响目标物的分子/离子状态，从而影响提取效率。故本研究分别考察了乙腈、乙腈+氨水、乙腈+甲酸和乙腈+乙酸对目标物的提取效率。发现乙腈中添加甲酸和乙酸会导致样品中DTPD和DPPD的回收率异常增高，添加氨水则可提高IPPD、CPPD和77PD等的回收率。这可能是因为PPDs含有2个亚氨基（-NH），其在酸性条件下发生质子化，不利于有机溶剂提取，而在碱性有机溶剂中，其主要以中性分子状态存在，有利于提取。进一步优化了氨水的含量，发现当添加氨水的体积分数为1%时，除77PD外，其他各目标物的回收率为63.0%~116.3%，进一步提高氨水的体积分数对目标物回收率影响不明显。故选用1%（体积分数）氨水乙腈为提取溶液。

PPDs及其醌类氧化物在氧气、光照等作用下可能发生降解，影响定量结果的准确性，故使用谷胱甘肽作为保护剂^［26］^。然而，也有研究指出，谷胱甘肽虽能减少6PPD氧化降解，但对其他潜在代谢产物的稳定性影响未知^［27］^。本文研究发现，提取液中添加20 μmol/L还原型谷胱甘肽可以提高IPPD、CPPD和7PPD等的回收率，进一步提高谷胱甘肽的浓度并不明显改善提取效果（图2）。因此，选择在提取液中添加20 μmol/L谷胱甘肽。

**图2:**
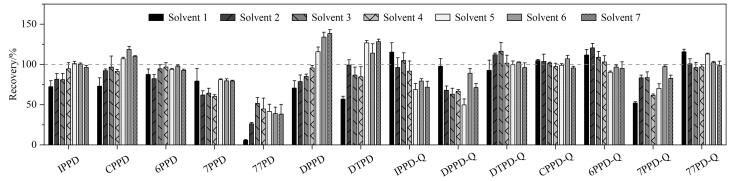
不同提取溶液对目标物回收率的影响（*n*=3）

#### 2.2.2 除水剂和净化剂

PM_2.5_基质复杂，可能对目标物的定量检测产生干扰。因此，有必要对提取液进行净化处理。近年有研究者将QuEChERS方法应用到PM_2.5_中有机物的提取，并取得了良好的效果^［28，29］^。本文拟采用QuEChERS方法进行净化。首先，比较了无水MgSO_4_和无水Na_2_SO_4_对目标物回收率的影响，发现二者对目标物回收率的影响无明显差异，但无水Na_2_SO_4_除水后提取液中存在悬浮物。进一步对比了不同用量无水MgSO_4_对回收率的影响，发现0.5 g MgSO_4_和1.0 g MgSO_4_对目标物回收率的影响差异不明显，但0.5 g MgSO_4_除水后净化液中存在少量悬浮物。其可能的原因是，石英滤膜在超声波局部的剪切力和冲击力作用下，发生表面或边缘微小的碎裂，造成石英颗粒或纤维脱落。而通常情况下，石英表面带负电荷^［30］^，由于静电排斥而形成不易沉降的悬浮物。依据舒尔策-哈代规则（Schulze-Hardy rule）^［31］^，所加电解质中带相反电荷的离子价数越高，聚沉能力越大。显然，Mg^2+^相较于Na^+^具有更强的电荷中和与颗粒聚沉能力。因此，添加MgSO_4_后的离心效果较佳。而1.0 g MgSO_4_相较于0.5 g具有更高的Mg^2+^浓度，因而离心效果更好。故使用1.0 g MgSO_4_作为除水剂。

QuEChERS方法中常用的净化剂包括C_18_和PSA等。分别考察了不同用量C_18_（50、100、150 mg）对目标物回收率的影响。发现DPPD-Q和7PPD-Q的回收率随C_18_用量的增加而增加，当用量达到150 mg时与100 mg时相比无明显变化，而77PD的回收率仍然偏低。因此，选择在100 mg C_18_净化剂基础上，考察添加不同用量PSA（50、100、150 mg）对目标物回收率的影响。发现添加100 mg PSA可使77PD的回收率趋于正常，继续增加其用量无明显改善（图3）。样品提取液经100 mg C_18_和100 mg PSA净化后，各目标物回收率范围为80.0%~117.0%。

**图3:**
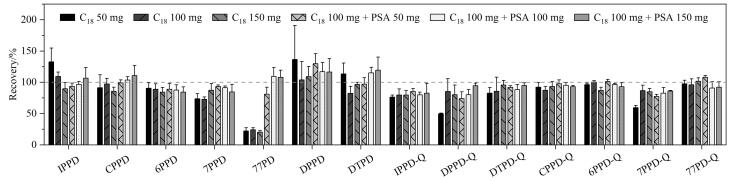
不同净化剂及用量对目标物回收率的影响（*n*=3）

#### 2.2.3 提取时间

为保证样品中目标物提取完全，比较了提取时间为30 min和60 min对目标物回收率的影响。结果如图4所示，提取时间30 min下，目标物的回收率为81.9%~105.4%。增加提取时间到60 min，部分目标物回收率有所提高，但均不存在显著性差异（*P*>0.05）。考虑到加标空白膜与实际PM_2.5_样品膜仍存在基质差异，分别在不同提取时间下检测了3份阳性样品。在30 min提取条件下，6PPD、IPPD和6PPD-Q的检测平均值分别为0.31、0.47和0.17 ng/mL；在60 min提取条件下，6PPD、IPPD和6PPD-Q的检测平均值分别为0.32、0.46和0.17 ng/mL。经成对样本进行T检验（95%置信区间），2组数据间无显著性差异，6PPD、IPPD和6PPD-Q的组间*t*值分别为-0.384（*P*=0.738）、0.565（*P*=0.629）和1.352（*P*=0.309），说明提取30 min可获得满意的提取效果。

**图4:**
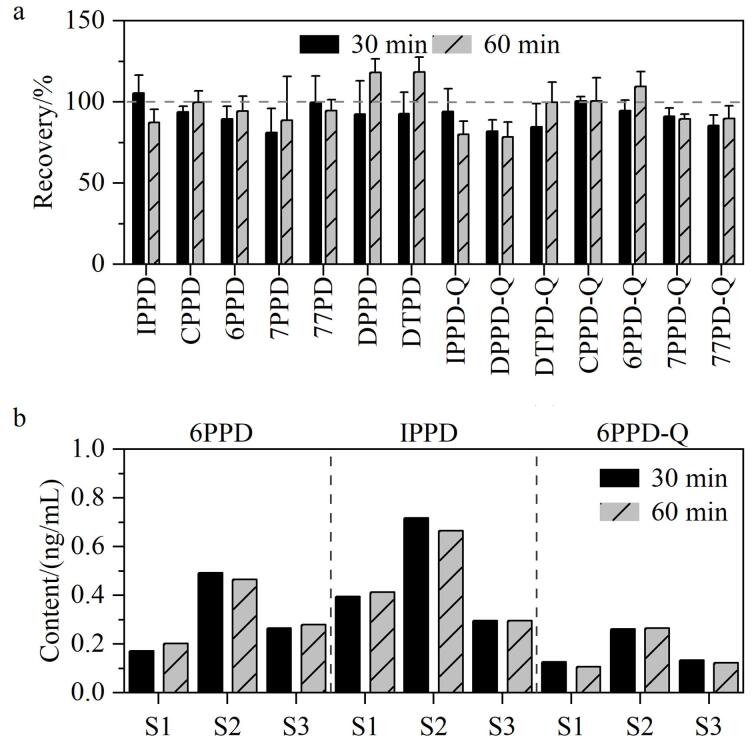
不同提取时间对目标物提取效率的影响

### 2.3 线性范围和检出限

按1.2节制备系列基质标准工作液，在已建立的仪器分析条件下进行测定。以目标物与对应内标的定量离子峰面积比为纵坐标（*y*），目标物与对应内标的质量浓度比为横坐标（*x*），绘制标准曲线。结果如表2所示，各目标物在0.05~20 ng/mL范围内线性关系良好，相关系数（*r*）均大于0.999。以3倍信噪比对应的浓度为检出限，10倍信噪比对应的浓度为定量限。本方法PPDs和PPD-Qs的仪器检出限（instrument detection limit，IDL）为0.001~0.02 ng/mL，仪器定量限（instrument quantification limit，IQL）为0.003~0.07 ng/mL。按100 L/min流量连续采集22 h计算，得到各目标物的方法检出限（method detection limit，MDL）为0.003~0.07 pg/m^3^，方法定量限（method quantification limit，MQL）为0.01~0.2 pg/m^3^（表2）。

**表2:** 方法的线性范围、线性方程、相关系数、方法检出限和方法定量限

Compound	Internal standard	Linear range/ （ng/mL）	Linear equation	*r*	MDL/ （pg/m^3^）	MQL/ （pg/m^3^）
IPPD	6PPD-d_5_	0.05-20	*y*=0.83*x*-0.0077	0.9990	0.07	0.2
CPPD	6PPD-d_5_	0.05-20	*y*=0.50*x*-0.0083	0.9991	0.01	0.04
6PPD	6PPD-d_5_	0.05-20	*y*=1.20*x*+0.011	0.9997	0.003	0.01
7PPD	6PPD-d_5_	0.05-20	*y*=0.75*x*-0.0015	0.9994	0.05	0.2
77PD	6PPD-d_5_	0.05-20	*y*=0.69*x*-0.0018	0.9998	0.04	0.2
DPPD	6PPD-d_5_	0.05-20	*y*=0.22*x*-0.0017	0.9994	0.02	0.1
DTPD	6PPD-d_5_	0.05-20	*y*=0.096*x*-0.00057	0.9999	0.04	0.1
IPPD-Q	6PPD-Q-d_5_	0.05-20	*y*=0.83*x*+0.012	0.9994	0.04	0.2
DPPD-Q	6PPD-Q-d_5_	0.05-20	*y*=0.40*x*+0.018	0.9993	0.02	0.07
DTPD-Q	6PPD-Q-d_5_	0.05-20	*y*=0.45*x*+0.0010	0.9994	0.01	0.03
CPPD-Q	6PPD-Q-d_5_	0.05-20	*y*=0.95*x*+0.011	0.9998	0.03	0.09
6PPD-Q	6PPD-Q-d_5_	0.05-20	*y*=0.72*x*-0.0065	0.9990	0.06	0.2
7PPD-Q	6PPD-Q-d_5_	0.05-20	*y*=0.42*x*+0.014	0.9991	0.06	0.2
77PD-Q	6PPD-Q-d_5_	0.05-20	*y*=1.70*x*+0.0026	0.9994	0.06	0.2

*y*： peak area ratio of analytes to internal standards； *x*： mass concentration ratio of analytes to internal standards.

### 2.4 基质效应

PM_2.5_基质复杂，含有多种金属元素、无机离子和有机化合物等^［16］^，可能造成基质增强或抑制效应，从而影响目标化合物的准确定量。本文采用空白基质匹配标准曲线的斜率与纯溶剂标准曲线的斜率的比值来计算各目标物的基质效应（ME）。一般认为，当ME为80%~120%时，基质效应较弱；否则为存在一定强度的基质效应^［24］^。如图5所示，样品溶液净化前，DPPD、DTPD存在较强的基质增强效应，7PPD-Q、77PD-Q和IPPD-Q等存在基质抑制效应。提取液经净化后，除IPPD-Q和7PPD-Q存在基质抑制效应外，其他各目标物的ME为81.7%~115.5%，均为弱基质效应。为降低ME的影响及准确定量，采用基质匹配内标法进行校正。

**图5:**
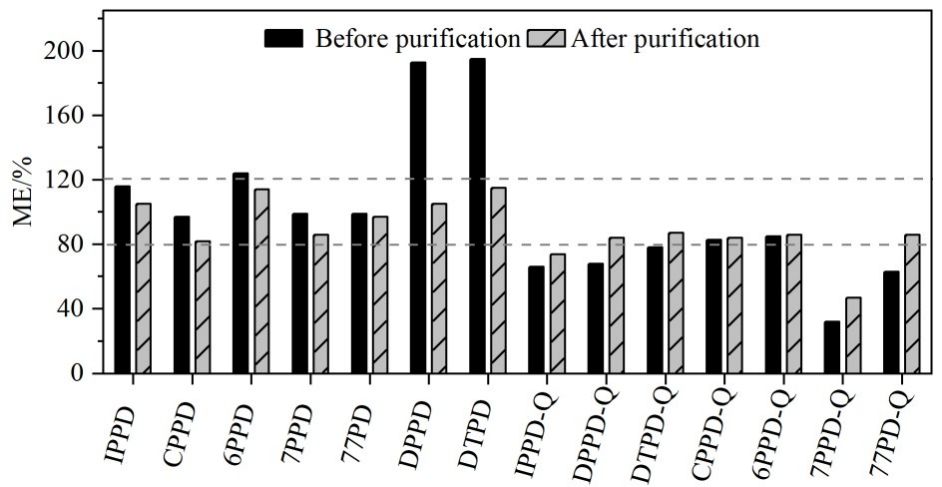
净化前后PPDs和PPD-Qs的基质效应对比

### 2.5 方法的准确度和精密度

分别向空白采样滤膜中添加40 μL质量浓度为1.0、10、100 ng/mL的目标物混合标准溶液，作为低、中、高3个水平的加标样品，每个水平的加标样品制备6份。结果显示，各目标物的回收率为66.3%~119.0%，相对标准偏差（RSD）为1.5%~13.2%（表3）。

**表3:** PPDs和PPD-Qs在低、中、高3个水平下的加标回收率及精密度（*n*=6）

Compound	Low		Medium		High	
	Recovery/%	RSD/%	Recovery/%	RSD/%	Recovery/%	RSD/%
IPPD	80.0	9.2	113.8	4.4	104.1	5.8
CPPD	73.8	9.3	108.2	3.4	104.2	7.0
6PPD	94.4	8.8	103.3	8.7	92.9	7.4
7PPD	98.0	12.4	117.1	4.9	119.0	3.3
77PD	113.8	4.0	84.0	1.5	73.9	3.7
DPPD	91.0	11.6	107.4	12.8	102.3	12.2
DTPD	115.3	10.5	114.1	12.5	111.2	4.9
IPPD-Q	78.2	7.0	98.1	3.3	94.3	7.9
DPPD-Q	107.0	12.3	76.5	11.0	66.3	5.9
DTPD-Q	102.6	5.4	117.5	8.5	95.0	3.8
CPPD-Q	83.0	12.5	109.0	7.3	100.2	6.0
6PPD-Q	87.0	11.9	112.0	12.5	101.6	4.8
7PPD-Q	74.5	13.2	111.4	7.6	86.5	8.4
77PD-Q	92.7	11.5	102.1	6.6	97.2	5.9

### 2.6 样品测定

采用本方法对包头市2025年1、2月的PM_2.5_样品进行检测。结果如表4所示，包头市PM_2.5_中7种PPDs均被检出，∑PPDs的质量浓度范围为2.26~251.2 pg/m^3^。除IPPD-Q和DTPD-Q未检出外，其余5种PPD-Qs均有检出，∑PPD-Qs的质量浓度范围为2.36~105.4 pg/m^3^。

**表4:** 包头市2025年1、2月的PM_2.5_中PPDs和PPD-Qs的检出率和质量浓度（*n*=28）

Compound	Detection rate/%	Measured values/（pg/m^3^）					
		Minimum	P_25_	Median	P_75_	Maximum	Mean
6PPD	100	0.07	10.6	20.0	33.0	99.0	28.3
IPPD	82	0.67	8.27	13.3	21.9	63.7	15.6
7PPD	50	0.48	0.48	1.03	11.1	16.9	5.81
DTPD	15	0.36	0.36	0.36	0.36	35.2	4.94
CPPD	9	0.12	0.12	0.12	0.12	5.03	0.42
77PD	8	0.38	0.38	0.38	0.38	10.1	1.34
DPPD	3	0.18	0.18	0.18	0.18	21.3	0.92
6PPD-Q	57	0.55	0.55	5.39	9.12	19.8	5.66
CPPD-Q	20	0.24	0.24	0.24	0.39	3.76	0.74
77PD-Q	16	0.67	0.67	0.67	0.67	13.4	2.85
DPPD-Q	14	0.24	0.24	0.24	0.24	60.9	4.91
7PPD-Q	3	0.67	0.67	0.67	0.67	7.58	0.93

### 2.7 与现有方法的比较

如表5所示，与现有文献方法相比较，本文建立的方法具有试剂消耗量少、提取时间短和方法检出限低的优点。

**表5:** 本方法与文献方法的比较

Number of target compounds	Solvent volume/mL	Redissolved volume/mL	Extraction time/min	Methods	MDLs/（pg/m^3^）	Ref.
1	11	0.5	90	UPLC-MS/MS	0.03	［^17^］
7	32	2	-	UHPLC-MS/MS	0.003-0.01^a^	［^18^］
10	15	0.1	45	UHPLC-MS/MS	0.02-0.3^a^	［^19^］
12	12	0.2	30	UPLC-MS/MS	0.04-0.3^b^	［^20^］
3	30	0.1	90	HPLC-Q-TOF-MS	-	［^22^］
14	10	0.2	30	HPLC-MS/MS	0.003-0.07， 0.001-0.02^a^， 0.01-0.2^b^	this study

a. instrument detection limit （IDL）， ng/mL； b. MQL； -： not given.

## 3 结论

本研究采用单一溶剂萃取法，将QuEChERS净化方法与HPLC-MS/MS技术相结合，建立了一种同时测定PM_2.5_中7种PPDs和7种PPD-Qs的方法。该方法具有试剂消耗量少、提取时间短、灵敏度高和准确性好的优点，可为PPDs和PPD-Qs的大气环境暴露监测提供技术参考。

## References

[1] Jiang N， Hao X X， Wang Z C， et al . J Hazard Mater， 2024， 476： 135122 38986411 10.1016/j.jhazmat.2024.135122

[2] Peng Z F， Hou S J， He Q Y， et al . Ecotox Environ Safe， 2025， 289： 117655 10.1016/j.ecoenv.2024.117655 39778316

[3] Geng N B， Hou S J， Sun S， et al . Environ Sci Technol， 2025， 59（6）： 3183 39927714 10.1021/acs.est.4c09519

[4] Zhu J Q， Guo R Y， Ren F F， et al . Sci Total Environ， 2024， 914： 170046 38218485 10.1016/j.scitotenv.2024.170046

[5] Wu W， Xu Q， Li J H， et al . Environ Pollut， 2024， 358： 124477 38950845 10.1016/j.envpol.2024.124477

[6] Hua X， Wang D Y . J Hazard Mater， 2023， 459： 132265 37595463 10.1016/j.jhazmat.2023.132265

[7] Tian Z Y， Zhao H Q， Peter K T， et al . Science， 2021， 371（6525）： 185 33273063 10.1126/science.abd6951

[8] He W M， Gu A H， Wang D Y . Sci Total Environ， 2023， 894： 164842 37336398 10.1016/j.scitotenv.2023.164842

[9] He W M， Chao J， Gu A H， et al . Sci Total Environ， 2024， 922： 171220 38412880 10.1016/j.scitotenv.2024.171220

[10] Li G， Dan L， Zhai W B， et al . Ecotoxicol Environ Saf， 2025， 301： 118494 40505274 10.1016/j.ecoenv.2025.118494

[11] Fang J C， Wang X X， Cao G D， et al . J Hazard Mater， 2024， 465： 133312 38147746 10.1016/j.jhazmat.2023.133312

[12] Ma C S， Liu Y X， Han B， et al . Environ Sci Technol， 2025， 59（3）： 1542 39810414 10.1021/acs.est.4c09276

[13] Wu X G， Hu J S， Yuan Z J， et al . J Hazard Mater， 2024， 480： 136176 39418905 10.1016/j.jhazmat.2024.136176

[14] Chen H F， Jin H B， Ren F F， et al . Environ Pollut， 2025， 378： 126489 40398798 10.1016/j.envpol.2025.126489

[15] Han M M， Xia K H， Xue Y， et al . Environ Sci Tech Let， 2025， 12（5）： 496

[16] Ma L X， Tran P T M， Balasubramanian R . Chemosphere， 2025， 372： 144103 39823957 10.1016/j.chemosphere.2025.144103

[17] Zhang Y J， Xu T T， Ye D M， et al . Environ Sci Tech Let， 2022， 9（5）： 420

[18] Zhang Y H， Xu C H， Zhang W F， et al . Environ Sci Technol， 2022， 56（11）： 6914 34551519 10.1021/acs.est.1c04500

[19] Cao G D， Wang W， Zhang J， et al . Environ Sci Technol， 2022， 56（7）： 4142 35316033 10.1021/acs.est.1c07376 PMC8988306

[20] Xia K H， Qin M， Han M M， et al . Environ Int， 2025， 197： 109329 39978217 10.1016/j.envint.2025.109329

[21] Wang W， Cao G D， Zhang J， et al . Environ Sci Technol， 2024， 58（13）： 5921 38512777 10.1021/acs.est.4c00027 PMC10993393

[22] Abudumutailifu M， Li C Z， Xiong H P， et al . ACS Earth Space Chem， 2025， 9（4）： 795 40671878 10.1021/acsearthspacechem.4c00291 PMC12262421

[23] Wang W， Cao G D， Zhang J， et al . Environ Sci Technol Lett， 2022， 9： 712

[24] Lu Z L Z， Deng F F， Bai Z J， et al . Chinese Journal of Chromatography， 2025， 43（9）： 1025 40910309 10.3724/SP.J.1123.2025.02010 PMC12412019

[25] Rudakov O B， Rudakova L V， Selemenev V F . J Anal Chromatogr Spectrosc， 2018， 1： 1. DOI： 10.24294/jacs.v1i2.883

[26] Zhu W L， Li F H， Liu X Y， et al . Journal of Instrumental Analysis， 2025， 44（8）： 1478

[27] Cheng J W， Zhu J S， Liu Y J， et al . Chinese Journal of Chromatography， 2025， 43（8）： 868

[28] Liu F， Tang B B， Kang Y H， et al . Journal of Hygiene Research， 2025， 54（6）： 995 41506995 10.19813/j.cnki.weishengyanjiu.2025.06.018

[29] Yan W Y， Wang C， Liu J， et al . Journal of Environmental and Occupational Medicine， 2024， 41（10）： 1087

[30] Liu C F . ［PhD Dissertation］. Huainan： Anhui University of Science and Technology， 2019： 29

[31] China National Committee for Terminology in Science and Technology . Chinese Terms in Chemistry. 2nd ed. Beijing： Science Press， 2016： 470

